# Association of dementia with the 28-day mortality of sepsis: an observational and Mendelian randomization study

**DOI:** 10.3389/fnagi.2024.1417540

**Published:** 2024-11-13

**Authors:** Ying Lan, Junchen Zhu, Peng Pu, Wentao Ni, Qilin Yang, Lvlin Chen

**Affiliations:** ^1^Department of Critical Care Medicine, Affiliated Hospital of Chengdu University, Chengdu, China; ^2^Department of Cardiology, The First Affiliated Hospital of Chongqing Medical University, Chongqing, China; ^3^Department of Pulmonary and Critical Care Medicine, Peking University People’s Hospital, Beijing, China; ^4^Department of Critical Care, The Second Affiliated Hospital of Guangzhou Medical University, Guangzhou, China

**Keywords:** dementia, sepsis, 28-day mortality, Mendelian randomization, MIMIC-IV

## Abstract

**Background:**

Observational research suggests that individuals with dementia who have sepsis face a higher likelihood of death. However, whether there is a causal relationship between the two remains unknown.

**Methods:**

We analyzed data from patients diagnosed with sepsis and dementia, extracted from the Medical Information Mart for Intensive Care IV (MIMIC-IV) database. To examine the correlation between dementia and 28-day mortality in sepsis, we utilized Cox proportional hazards models. Following this, we performed a Mendelian randomization (MR) study with two samples to investigate the potential link between dementia and mortality within 28 days in sepsis.

**Results:**

This study included a total of 22,189 patients diagnosed with sepsis, among whom 1,346 cases (6.1%) had dementia. After adjusting for multiple confounding factors, dementia was associated with an increased risk of 28-day mortality in sepsis (HR = 1.25, 95% CI = 1.12–1.39, *p* < 0.001). In the MR analysis, there appeared to be a causal relationship between genetically predicted dementia with Lewy bodies (DLB) (OR = 1.093, 95% CI = 1.016–1.177, *p* = 0.017) and 28-day mortality in sepsis. However, there was no evidence of causality between any dementia (OR = 1.063, 95% CI = 0.91–1.243, *p* = 0.437), Alzheimer’s disease (AD) (OR = 1.126, 95% CI = 0.976–1.299, *p* = 0.103), vascular dementia (VD) (OR = 1.008, 95% CI = 0.93–1.091, *p* = 0.844), and the risk of 28-day mortality in sepsis.

**Conclusion:**

In the observational analysis, dementia was associated with an increased risk of 28-day mortality in septic patients. However, in the MR analysis, only DLB was associated with increased 28-day mortality in septic patients, with no observed correlation for other dementia subtypes.

## 1 Introduction

Globally, dementia ranks as the seventh main contributor to mortality and is a significant factor in disability (World Health Organization, 2023). In 2019, there were an estimated 55.2 million dementia patients worldwide, with societal costs estimated at $1.3134 trillion annually. By 2050, it is projected that the prevalence of dementia will rise to 130 million cases (World Health Organization, 2023). The enormous cost of dementia globally imposes significant pressure on societies and healthcare systems.

Sepsis, a condition where the body’s response to infection becomes uncontrolled, can lead to life-threatening organ dysfunction (Angus and van der Poll, 2013). It is one of the major health issues worldwide. Sepsis is responsible for around 20% of worldwide fatalities, as per statistical data (Rudd et al., 2020). According to previous research (Bouza et al., 2019; Shen et al., 2012), dementia has been found to significantly impact the treatment and prognosis of septic patients. Dementia patients have a 50% risk of developing severe sepsis (Shen et al., 2012). According to our understanding, there is only one research that has documented an inverse association between dementia and in-patient fatality among septic elderly individuals (Bouza et al., 2019). Nevertheless, the current understanding of the association between dementia and 28-day mortality in sepsis is ambiguous. Therefore, we extracted information from the MIMIC-IV database regarding dementia and sepsis patients to investigate the relationship between dementia and 28-day mortality in sepsis. However, due to the limitations of retrospective studies, such as potential confounding factors and selection bias, conclusions regarding the association between dementia and sepsis outcomes may be affected (Bouza et al., 2019; Shen et al., 2012). In order to investigate the correlation between dementia and 28-day death in sepsis, we conducted a Mendelian randomization (MR) study to address the constraints of retrospective investigation.

MR assesses causal links between exposure and outcome by integrating genetic instrumental variables (IVs), like single nucleotide polymorphisms (SNPs) (Sekula et al., 2016). Given that IVs are not influenced by other forms and are inherited randomly, MR analysis can effectively minimize the impact of confounding factors and decrease the likelihood of reverse causality (Verduijn et al., 2010). Through implementing MR studies, our aim was to evaluate the relationship between dementia and 28-day mortality in sepsis.

## 2 Materials and methods

### 2.1 Overall study design

This study consists of two parts. Using data from MIMIC-IV 2.2, we examined the correlation between dementia and mortality rates at both 28 days and in the long term for sepsis patients. For the second segment, we performed MR investigation by utilizing summary statistics information from genome-wide association studies (GWAS) to assess the causal influence of genetically predicted dementia on the mortality within 28 days of sepsis.

### 2.2 Observational study

#### 2.2.1 Data source

The research employed the electronic dataset sourced from the Medical Information Mart for Intensive Care-IV (MIMIC-IV 2.2), which is a database that is openly accessible and available for free (Johnson et al., 2023). Between 2008 and 2019, the dataset includes medical documents of individuals who were hospitalized in the intensive care wards at Beth Israel Deaconess Medical Center. The authors (YL and QY) were granted access to the database and responsible for data extraction, with certification numbers 50391342 and 7634793. Because the patient health information in this database is anonymized, the study was exempt from review by the Institutional Review Board.

#### 2.2.2 Patient population

This study included adult ICU patients meeting the criteria for sepsis 3.0, defined as suspected infection and SOFA scores ≥ 2 (Singer et al., 2016). The method for identifying sepsis patients based on the sepsis 3.0 diagnostic criteria was consistent with previous studies and was obtained from the MIMIC database (Hu et al., 2023; see Supplementary Method A). The identification of dementia was accomplished by utilizing diagnostic codes from ICD-9-CM and ICD-10-CM (refer to Supplementary Method B). Exclusion criteria: (1) Age under 18 years; (2) For patients who have been admitted to the ICU multiple times due to sepsis, only the data from their first admission will be extracted. Our study was reported following the guidelines outlined in the RE porting of studies Conducted using Observational Routinely-collected health Data (RECORD) Statement (Benchimol et al., 2015).

#### 2.2.3 Variable extraction

The study involved extracting information through the execution of SQL queries using Postgres SQL software (version 13.7.2) and Navicat Premium software (version 16), with the assistance of Structured Query Language (SQL). Data obtained from the MIMIC-IV database within the first 24 h of ICU admission included age, sex, body mass index (BMI), race, marital status, smoking status, comorbidities, SOFA score, and Charlson Comorbidity Index. Other relevant data encompassed vital signs, laboratory tests, treatments, and clinical outcomes. If a variable was recorded more than once, we used the worst value within 24 h. Consensus has not been reached regarding the missing value criterion for excluding variables from analysis. Referring to previous literature (Zhang et al., 2019; Zheng et al., 2023), in our study, the threshold was set at 60% (see Supplementary Table 1). In the study, multiple imputation was used to address missing data for covariates. Five imputed datasets were created and analyzed together.

#### 2.2.4 Primary exposure and outcomes

The primary exposure in this study was dementia, encompassing all dementia data extractable from the database. The primary outcome was the 28-day mortality. Secondary outcomes included in-hospital mortality, 90-day mortality, and 1-year mortality. The time variable starts on the day of ICU admission. Mortality information for discharged patients was obtained from the United States Social Security Death Index.

#### 2.2.5 Statistical analysis

The study population was divided into dementia patients and non-dementia patients. Mean (standard deviation) or median [interquartile range (IQR)] were used to present continuous variables, whereas counts (percentages) were used to present categorical variables. Categorical variables were analyzed using either the Chi-square test or Fisher’s exact test, while intergroup comparisons of continuous variables were conducted using either Student’s *t*-test or Mann-Whitney U test.

Kaplan–Meier survival curves for the 28-day mortality were plotted for both groups, and the Log-Rank test was used for comparison. A multivariable Cox proportional hazards model was used to estimate the impact of dementia on the 28-day mortality. Three models were employed to adjust for potential confounders: Model 1: Unadjusted for any confounders. Model 2: Adjusted for age, sex, BMI, smoking, race, and marital status. Model 3: Building upon Model 2, additional adjustment was made for confounders including heart rate, mean arterial pressure, SPO2, hemoglobin, platelets, creatinine, WBC, glucose, potassium, sodium, lactate, SOFA score, Charlson Comorbidity Index, mechanical ventilation (MV) use (first 24 h), and vasopressor use (first 24 h). Subgroup analyses were conducted including age, sex, BMI, SOFA and vasopressor use. Additionally, we performed sensitivity analyses by excluding missing data for covariates and analyzed the complete dataset.

All statistical analyses were performed using R (version 4.2.2) and Free Statistics software version 1.9.1 (Beijing Free Clinical Medical Technology Co., Ltd.). A significance level of *P* < 0.05 was considered statistically significant.

### 2.3 Mendelian randomization

#### 2.3.1 Study design and genetic instrument selection

The overall design process of the MR analysis is illustrated in Supplementary Figure 1. We used summary statistics data from publicly accessible GWAS sources, ensuring that all studies had obtained ethical approval from their respective institutional review boards and written informed consent from participants. The genetic variants used in MR analysis need to satisfy three assumptions: (1) Genetic variants are presumed to correlate with exposure levels. (2) They must not correlate with confounding factors. (3) Genetic variants solely influence outcomes through exposure (Evans and Davey Smith, 2015). Based on these assumptions, we opted for single nucleotide polymorphisms (SNPs) as instrumental variables (IVs), requiring significant association with the exposure (*P* < 5 × 10^–8^). To ensure the independence of each SNP, the LD threshold was set at *r*^2^ < 0.001, with a genetic distance of 10,000 kb. If following the above criteria, the number of SNPs contained in DLB and VD was too few. Therefore, for DLB and VD, we adopted a threshold of *P* < 5 × 10^–6^. To address weak instrument bias, we calculated the F statistic. F statistic > 10 indicates that the estimated effect between IVs and exposure is robust (Burgess and Thompson, 2011). To eliminate potential confounding variables, we used PhenoScanner to search for each included SNP and excluded those closely associated with confounders. Finally, the remaining selected SNPs served as instrumental variables for subsequent analysis. This study adheres to the STROBE-MR guidelines (Skrivankova et al., 2021).

#### 2.3.2 Data sources for exposures and outcomes

Exposure included any dementia and three subtypes of dementia, including AD, VD, and DLB. Due to the scarcity of FTD genetic variants, we did not include them in the analysis. GWAS data for any form of dementia and VD were sourced from the FinnGen consortium, AD’s GWAS summary statistics data came from the International Genomics of Alzheimer’s Project (IGAP), and DLB’s GWAS data originated from another independent multicenter study. The outcome was 28-day death in sepsis, established by the UK Biobank, comprising 486,484 Europeans (1,896 cases and 484,588 controls) and 12,243,487 SNPs. In Supplementary Table 4, we systematically summarized the GWAS data characteristics for exposure and outcome.

#### 2.3.3 Statistical analysis

The impact of the exposure on each IV outcome was estimated using the Wald ratio, and then the effect sizes of each IV were combined using the inverse variance weighting (IVW) method with random-effects IVW (Hemani et al., 2018). Furthermore, we utilized the weighted median (WM) technique and the MR-Egger approach as complements to IVW. Cochrane’s Q statistic was utilized to assess heterogeneity. Horizontal pleiotropy was detected using the MR-Egger intercept. Outlier detection using MR-PRESSO identified anomalies, which were subsequently removed. All statistical analyses were conducted using R version 4.2.2^1^ with the Mendelian randomization, Two Sample MR, and MR-PRESSO packages.

## 3 Results

### 3.1 Observe study

#### 3.1.1 Characteristics

**FIGURE 1:**
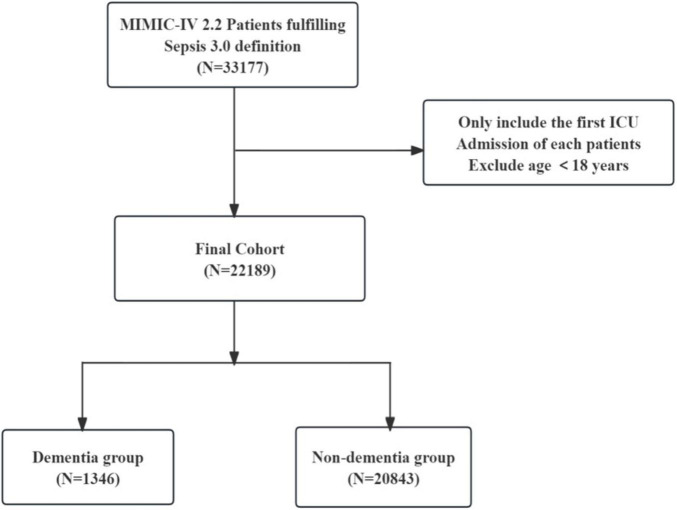
Flowchart of subject selection.

After inclusion and exclusion criteria, the final analysis included a total of 22,189 sepsis patients (Figure 1). Among them, 1,346 cases (6.1%) had dementia. Based on whether dementia was present or not, the basic characteristics of the study subjects are summarized in Table 1. Overall, compared to the non-dementia group, patients in the dementia group were older (65.4 ± 16.3 vs. 82.4 ± 9.2 years), had higher Charlson scores (5.7 ± 2.9 vs. 7.2 ± 2.2), and lower BMI (29.1 ± 7.6 vs. 25.7 ± 5.8). Interestingly, the dementia group had lower SOFA scores (5.8 ± 3.3 vs. 5.4 ± 2.8), lower rates of MV on the first day (48.1 vs. 29.9%), and lower rates of vasopressor use (45.5 vs. 33.9%).

**TABLE 1:** Baseline characteristics of participants.

Variables	All patients (*n* = 22,189)	Non-dementia (*n* = 20,843)	Dementia (*n* = 1,346)	*P*-value
Age, [years, mean (SD)]	66.5 ± 16.4	65.4 ± 16.3	82.4 ± 9.2	< 0.001
Male, [*n* (%)]	12,799 (57.7)	12,166 (58.4)	633 (47)	< 0.001
BMI, [kg/m^2^, mean (SD)]	29.0 ± 7.6	29.1 ± 7.6	25.7 ± 5.8	< 0.001
smoking, [*n* (%)]	5,944 (26.8)	5,762 (27.6)	182 (13.5)	< 0.001
married, [*n* (%)]	9,903 (44.6)	9,425 (45.2)	478 (35.5)	< 0.001
Race, [*n* (%)]				0.885
White	14,860 (67.0)	13,961 (67)	899 (66.8)	
No-white	7,329 (33.0)	6,882 (33)	447 (33.2)	
Heart rate, [bpm, mean (SD)]	105.9 ± 21.0	106.0 ± 20.9	104.4 ± 21.9	0.006
MAP, [mmHg, mean (SD)]	56.8 ± 13.2	56.8 ± 13.3	55.4 ± 12.9	< 0.001
SpO2, [%, mean (SD)]	91.3 ± 7.1	91.4 ± 7.1	90.3 ± 7.4	< 0.001
WBC, [10^9^/L, mean (IQR)]	13.8 (9.9, 18.7)	13.8 (9.9, 18.7)	13.4 (9.9, 18.3)	0.129
Hemoglobin, [g/L, mean (IQR)]	9.8 (8.4, 11.4)	9.8 (8.3, 11.3)	10.2 (8.6, 11.5)	< 0.001
Platelet, [10^9^/L, mean (IQR)]	159.0 (110.0, 223.0)	157.0 (109.0, 222.0)	180.0 (133.0, 237.0)	< 0.001
Glucose, [mg/L, mean (IQR)]	112.0 (94.0, 135.0)	112.0 (95.0, 134.5)	112.0 (93.0, 136.0)	0.47
Creatinine, [mg/dL, mean (IQR)]	1.1 (0.8, 1.8)	1.1 (0.8, 1.8)	1.3 (0.9, 1.9)	< 0.001
Sodium, [mmol/L, mean (IQR)]	137.0 (134.0, 140.0)	137.0 (134.0, 139.0)	139.0 (136.0, 142.0)	< 0.001
Potassium, [mmol/L, mean (IQR)]	3.9 (3.5, 4.3)	3.9 (3.5, 4.3)	3.8 (3.5, 4.3)	0.008
Lactate, [mmol/L, mean (IQR)]	2.3 (1.5, 3.6)	2.3 (1.5, 3.6)	2.1 (1.4, 3.7)	0.045
CCI, [mean (SD)]	5.8 ± 2.9	5.7 ± 2.9	7.2 ± 2.2	< 0.001
SOFA score, [mean (SD)]	5.7 ± 3.3	5.8 ± 3.3	5.4 ± 2.8	< 0.001
MV use (1st 24 h), [*n* (%)]	10,435 (47.0)	10,032 (48.1)	403 (29.9)	< 0.001
Vasopressor use (1st 24 h), [*n* (%)]	9,930 (44.8)	9,474 (45.5)	456 (33.9)	< 0.001

BMI, body mass index; MAP, mean arterial pressure; WBC, white blood cell; BUN, blood urea nitrogen; CCI, Charlson Comorbidity Index; SOFA, Sequential Organ Failure Assessment; MV, mechanical ventilation.

#### 3.1.2 Primary outcome

The relationship between dementia and 28-day mortality in sepsis is illustrated in Supplementary Figure 2. Kaplan–Meier survival curves indicate that the 28-day mortality for sepsis in the dementia group and non-dementia group were 30.5 and 17.6%, respectively (Log-Rank test: *P* < 0.0001). In the original model, the dementia group showed a higher 28-day mortality related to sepsis (HR = 1.84, 95% CI = 1.66–2.04, *p* < 0.001) compared to the non-dementia group. After accounting for all covariates in Model 3 using multivariable Cox regression analysis, dementia continued to be linked to a higher risk of mortality within 28 days in sepsis. The findings remained consistent, indicating an increased risk with an HR of 1.25 (95% CI = 1.12–1.39, *p* < 0.001) as shown in Table 2. We also conducted subgroup analyses based on age (< 80 years, ≥ 80 years), sex, BMI (< 25, ≥ 25), SOFA score (< 6, ≥ 6), and first-day vasopressor use (yes or no). All subgroup analyses yielded consistent results (Supplementary Figure 3). Additionally, in patients with a complete dataset, the association between dementia and the risk of mortality from sepsis at 28 days, 90 days, and 1 year was consistent with the core results (Supplementary Table 3).

**TABLE 2:** The relationship between dementia and 28-day mortality of sepsis.

	Deaths, *n* (%)		HR (95% CI)	*P*-value
	**Dementia**	**Non-dementia**		
Model 1	411 (30.5)	3,675 (17.6)	1.84 (1.66∼2.04)	< 0.001
Model 2	411 (30.5)	3,675 (17.6)	1.23 (1.11∼1.37)	< 0.001
Model 3	411 (30.5)	3,675 (17.6)	1.25 (1.12∼1.39)	< 0.001

Model 1 was an unadjusted. Model 2 adjusted for age, sex, BMI, smoking, race, and married; Model 3: Model 2+ heart rate, mean arterial pressure, SPO2, hemoglobin, platelets, creatinine, WBC, glucose, potassium, sodium, lactate, SOFA, Charlson Comorbidity Index, MV use (1st 24 h), Vasopressor use (1st 24 h). CI, confidence interval; HR, hazard ratio; BMI, body mass index; WBC, white blood cell; SOFA, Sequential Organ Failure Assessment; MV, mechanical ventilation.

#### 3.1.3 Second outcome

**FIGURE 2:**
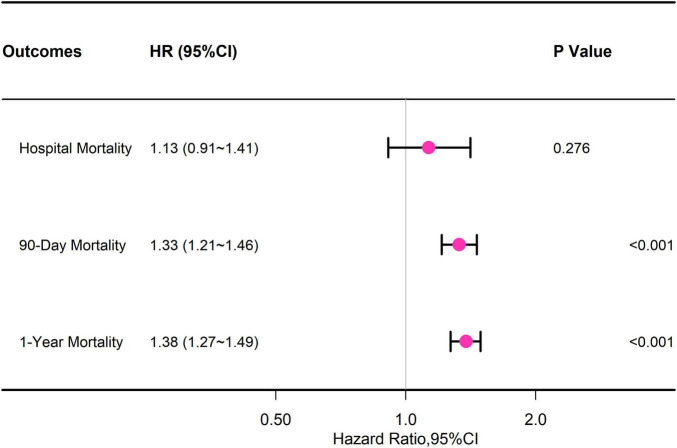
Association between dementia and secondary outcomes.

Upon accounting for all covariates, the Cox regression analysis demonstrated a correlation between dementia and increased 90-day mortality (HR = 1.33, 95% CI = 1.21–1.46, *p* < 0.001) as well as 1-year mortality (HR = 1.38, 95% CI = 1.27–1.49, *p* < 0.001). Nevertheless, sepsis did not show any connection with in-hospital death caused by dementia (HR = 1.13, 95% CI = 0.91–1.41, *p* = 0.276) (Figure 2 and Supplementary Table 2).

### 3.2 Mendelian randomization

#### 3.2.1 Selection of IVs

In this MR analysis, we obtained 11 SNPs associated with any dementia, 18 SNPs associated with AD, 21 SNPs associated with VD, and 19 SNPs associated with DLB. For exposure, SNPs obtained for any dementia and AD satisfied the currently acknowledged genome-wide exposure significance criteria (*p* < 5 × 10^–8^, *R*^2^ < 0.001, kb = 10,000). VD and DLB extracted fewer SNPs under the above criteria, thus selecting a threshold of *p* < 5 × 10^–6^. Hence, the robustness of these SNPs was evaluated by calculating F-statistics, which all exhibited significant power (F-statistics > 10 for all; Supplementary Tables 6, 7). MR-PRESSO did not identify any outliers that needed to be excluded. Supplementary Tables 5–8 provide information on the IVs for dementia and its related subcategories.

#### 3.2.2 MR analysis of two samples

**FIGURE 3:**
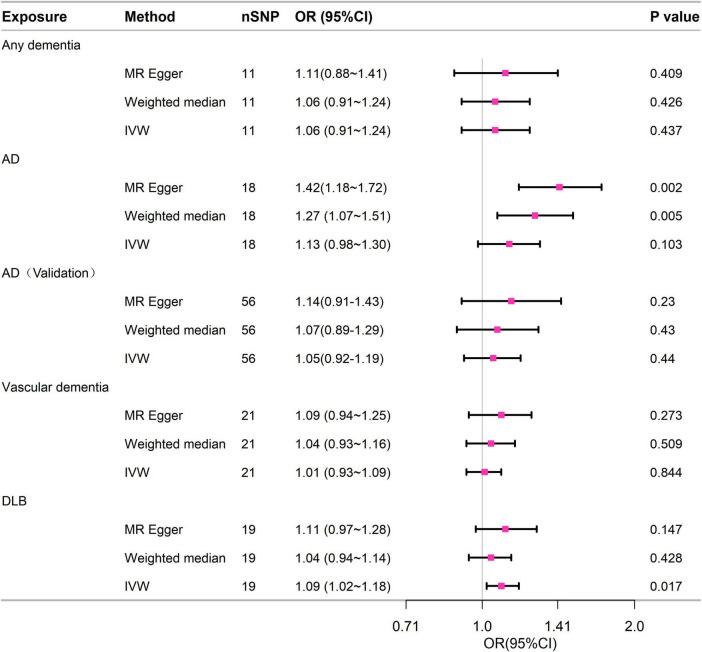
Forest plot of the MR study investigating the effect of dementia on 28-day mortality of sepsis. IVW, inverse variance weighting; AD, Alzheimer’s disease; CI: 95% confidence interval. OR, odds ratio.

Figure 3 contained the statistical outcomes regarding the association between dementia and its subtypes with the risk of 28-day mortality in sepsis. According to IVW analysis, genetically predicted DLB (OR = 1.093, 95% CI = 1.016–1.177, *p* = 0.017) was significantly linked to 28-day mortality in sepsis. The findings from MR-Egger and Mendelian Weighted (MW) were also consistent. However, no causal relationship was detected between dementia (OR = 1.063, 95% CI = 0.91–1.243, *p* = 0.437), AD (OR = 1.126, 95% CI = 0.976–1.299, *p* = 0.103), VD (OR = 1.008, 95% CI = 0.93–1.091, *p* = 0.844), and 28-day mortality in sepsis. Cochrane’s Q test in sensitivity analysis indicated no heterogeneity across all analyses. Moreover, except for AD, in other analyses, the *P*-values from the MR-Egger intercept tests were > 0.05, indicating no horizontal pleiotropy (Supplementary Table 9).

## 4 Discussion

In this study, we thoroughly investigated the relationship between dementia and 28-day mortality in sepsis through both observational analysis and MR studies. In the observational study, following adjustments for confounding factors, dementia showed an increased risk of 28-day mortality in sepsis. The MR study results showed that genetically predicted DLB was causally associated with the risk of 28-day mortality in sepsis. However, no evidence of causality was observed between any dementia and its subtypes and 28-day mortality in sepsis.

The risk of death in patients admitted to the hospital for acute illnesses is heightened by dementia (Morrison and Siu, 2000; Sampson et al., 2009). Likewise, individuals with dementia have a higher likelihood of developing sepsis (Janbek et al., 2021; Liao et al., 2015; Shen et al., 2012). Nevertheless, the connection between dementia and mortality risk in sepsis remains uncertain, as there is a scarcity of research on this subject. A retrospective study conducted on the Spanish population (Bouza et al., 2019) demonstrated an independent association between dementia presence and increased in-hospital mortality among sepsis patients. However, Shen et al. (2012) found in their study in Taiwan that dementia was not associated with in-hospital mortality in the overall population of hospitalized patients after life support therapy was controlled. In our retrospective analysis of MIMIC-IV data, we observed a significant association between dementia and higher 28-day mortality as well as long-term mortality in sepsis patients, but no association with in-hospital mortality. What sets our study apart was the innovative extension to a specific population within the ICU. Additionally, it marks the first attempt to assess long-term mortality risk among sepsis patients. Furthermore, in our cohort, dementia patients were older and had higher Charlson comorbidity scores, which was consistent with the literature (Angus and van der Poll, 2013; Bouza et al., 2016; Martin et al., 2006). Age and the presence of comorbidities were initially linked to a heightened risk of mortality in sepsis (Lagu et al., 2012; Pisani et al., 2005). Nevertheless, upon adjusting for confounding factors such as age, organ comorbidities, and organ dysfunction, the findings remained consistent.

Based on the aforementioned findings from the observational study, it was evident that dementia heightened the likelihood of 28-day mortality in sepsis. Nevertheless, this connection was frequently entangled with variables like disease severity, concurrent conditions, age, and the utilization of life-sustaining interventions. Therefore, to further mitigate confounding and bias, we conducted two-sample MR analysis. The findings suggested a causal link between genetically predicted DLB and elevated 28-day mortality risk in sepsis. However, there was no causal relationship found between any dementia, AD, VD, and 28-day mortality in sepsis. This suggests that while dementia overall may not universally increase the short-term mortality risk in sepsis patients, LBD may be a specific risk factor.

To our surprise, this study using MR revealed compelling evidence indicating a causal link between LBD and the likelihood of sepsis-related mortality within 28 days. Unfortunately, due to limitations of the MIMIC database, accurate data on LBD were not available for real-world validation. Based on our current understanding, LBD ranks as the second most prevalent form of neurodegenerative dementia (Taylor et al., 2020). Systematic reviews report that LBD accounts for 3 to 7% of dementia cases (Hogan et al., 2016). Compared to AD, cognitive decline progresses more rapidly, and the prognosis is worse in LBD (Oesterhus et al., 2014). In our analysis, we utilized the largest LBD GWAS to date, which comprised 2,591 LBD cases and 4,027 neurologically healthy individuals (Chia et al., 2021). Due to underdiagnosis or misdiagnosis of LBD in clinical settings, the sample size of LBD GWAS is relatively small, potentially leading to statistical power issues (Guo et al., 2022). Subsequent efforts are needed to expand the sample size for validation. Anyway, this inspires us to pay more attention to LBD in patients with sepsis.

The increased sepsis-related mortality in dementia patients can be explained by several mechanisms. Firstly, dementia is often associated with significant cholinergic dysfunction (Jorfi et al., 2023). The cholinergic system, through vagal nerve signaling, plays a crucial role in regulating inflammatory responses (Han et al., 2017). The failure of this mechanism impairs the patient’s ability to manage the systemic inflammatory response triggered by sepsis, leading to exacerbated organ dysfunction and increased mortality risk. Secondly, dementia patients frequently exhibit dysbiosis of the gut microbiota (Qian et al., 2022), which not only intensifies neural damage but also exacerbates the systemic inflammatory response in sepsis, resulting in adverse outcomes. Lastly, immune dysregulation in dementia patients increases susceptibility to infections, particularly aspiration pneumonia, and cognitive decline complicates the treatment of pulmonary infections (Bail et al., 2013; Graversen et al., 2021). Recent studies have shown significant alterations in peripheral cytokines (e.g., IL-1, IL-6) and lymphocyte subpopulations in DLB (Amin et al., 2022), but whether these changes indicate immune suppression or are associated with sepsis mortality risk requires further investigation.

This study has several limitations in observational research. The MIMIC database originates from a research institution that is solely based on one center, potentially restricting the applicability of the study results. Additionally, because of constraints in the database, we couldn’t distinguish between the particular forms of dementia. As a result, we couldn’t conduct a more in-depth examination of the mortality risk linked to various types of dementia and sepsis. Furthermore, certain data such as advance directives, socioeconomic status, and additional variables were not incorporated into the database. Additionally, the MR analysis has certain constraints. Horizontal pleiotropy is a common concern in all MR studies, to begin with. There is a possibility that our research still contains some hidden pleiotropy. Additionally, because there were only a few SNPs that met the criteria for dementia inclusion, we made slight adjustments to the selection criteria, potentially leading to the presence of some false positives. Additionally, our magnetic resonance study was carried out on individuals with European heritage, necessitating additional investigation to evaluate its applicability to diverse populations.

## 5 Conclusion

Observational findings suggested a correlation between dementia and 28-day mortality in sepsis, while MR analysis indicated a causal link only with LBD. Other types of dementia did not show evidence of causality.

## Data Availability

The datasets presented in this study can be found in online repositories. The names of the repository/repositories and accession number(s) can be found in this article/Supplementary material.

## Footnotes

1
https://www.r-project.org/
