# Supplementary material for: Association of dementia with the 28-day mortality of sepsis: an observational and Mendelian randomization study
Source: Front Aging Neurosci. 2024 Nov 13;16:1417540. doi: 10.3389/fnagi.2024.1417540 (PMC11599188; doi:10.3389/fnagi.2024.1417540)
Supplement: Supplementary file 1 [file Data_Sheet_1.docx]

Supplementary Material

**Additional methods:**

1. **Definition of “sepsis”**

Sepsis was defined based on previously described methods, including (1) Clinical suspicion of infection as determined by the earlier timestamp of antibiotics administration, and cultures within a certain timeframe. If antibiotics were given first, then the cultures must have been obtained within 24 hours. If cultures were obtained first, then antibiotics must have been subsequently ordered within 72 hours. (2) The occurrence of end-organ damage as identified by a two-point deterioration in SOFA score. (3) The onset time of sepsis is the earlier of $t_{\mathrm{suspicion}}$ and $t_{\mathrm{SOFA}}$ as long as $t_{\mathrm{SOFA}}$ occurs no more than 48 hours before or 24 hours after $t_{\mathrm{suspicion}}$; otherwise, the patient is not marked as a sepsis patient. Specifically, if $t_{\mathrm{suspicion}}$ -48 ≤ $t_{\mathrm{SOFA}}$ ≤ $t_{\mathrm{suspicion}}$ +24, then $t_{\mathrm{sepsis}}$ = min ($t_{\mathrm{suspicion}}$, $t_{\mathrm{SOFA}}$) (1-3).

1. **Dementia codes from ICD-9-CM to ICD-10-CM.**

- ICD-9-CM codes

2900, 2903, 29010, 29011, 29012, 29013, 29020, 29021, 29040, 29041, 29042, 29043, 29410, 29411;

- ICD-10-codes

F0150, F0151, F0280, F0281, F0390, F0391, G300, G301,G308,G309,G311

**Reference**

1. Yang M, Liu C, Wang X, Li Y, Gao H, Liu X, et al. An Explainable Artificial Intelligence Predictor for Early Detection of Sepsis. Crit Care Med. 2020;48(11):e1091-e6.

2. Reyna MA, Josef CS, Jeter R, Shashikumar SP, Westover MB, Nemati S, et al. Early Prediction of Sepsis From Clinical Data: The PhysioNet/Computing in Cardiology Challenge 2019. Crit Care Med. 2020;48(2):210-7.

3. Hu W, Chen H, Ma C, Sun Q, Yang M, Wang H, et al. Identification of indications for albumin administration in septic patients with liver cirrhosis. Crit Care. 2023;27(1):300.

**Table S1: Missing rate for demographics and clinical variables extracted from the database during the observation period.**

|  | Percent of missing (%) |
| --- | --- |
| Age | 0 |
| Sex | 0 |
| BMI | 38.8 |
| Smoking | 0 |
| Heart rate | 0.1 |
| MAP | 0.1 |
| SpO2 | 0.1 |
| WBC | 0.2 |
| Hemoglobin | 0.2 |
| Platelet | 0.2 |
| Glucose | 0.6 |
| Creatinine | 0.1 |
| Sodium | 0.2 |
| Potassium | 0.2 |
| Lactate | 34.1 |
| CCI | 0 |
| SOFA score | 0.1 |
| MV use (1^st^ 24 h) | 0 |
| Vasopressor use (1^st^ 24 h) | 0 |

Abbreviation: BMI, body mass index; MAP, mean arterial pressure; WBC, white blood cell; CCI, Charlson Comorbidity Index; SOFA, Sequential Organ Failure Assessment; MV, mechanical ventilation.

**Table S2 Association between dementia and secondary outcomes.**

|  | **Deaths, n. (%)** | | **HR (95%CI)** | **P-value** |
| --- | --- | --- | --- | --- |
|  | **Dementia** | **Non-dementia** |  |  |
| **Hospital mortality** | | | | |
| Model 1 | 3105 (14.9) | 264 (19.6) | 1.39 (1.23~1.58) | <0.001 |
| Model 2 | 3105 (14.9) | 264 (19.6) | 1.15 (0.96~1.39) | 0.127 |
| Model 3 | 3105 (14.9) | 264 (19.6) | 1.13 (0.91~1.41) | 0.276 |
| **90-day mortality** | | | | |
| Model 1 | 580 (43.1) | 4857 (23.3) | 2.05 (1.88~2.23) | <0.001 |
| Model 2 | 580 (43.1) | 4857 (23.3) | 1.33 (1.22~1.46) | <0.001 |
| Model 3 | 580 (43.1) | 4857 (23.3) | 1.33 (1.21~1.46) | <0.001 |
| **1-year mortality** | | | | |
| Model 1 | 6395 (30.7) | 774 (57.5) | 2.21 (2.06~2.39) | <0.001 |
| Model 2 | 6395 (30.7) | 774 (57.5) | 1.41 (1.3~1.52) | <0.001 |
| Model 3 | 6395 (30.7) | 774 (57.5) | 1.38 (1.27~1.49) | <0.001 |

Model 1 was an unadjusted model. Model 2 adjusted for age, sex, BMI, smoking, race, and married; Model 3: Model 2+ heart rate, mean arterial pressure, SpO₂, hemoglobin, platelets, creatinine, WBC, glucose, potassium, sodium, lactate, SOFA, Charlson comorbidity index, MV use (1st 24 h), Vasopressor use (1st 24 h). Abbreviations: CI, confidence interval; HR, hazard ratio; BMI, body mass index; WBC, white blood cells; SOFA, Sequential Organ Failure Assessment; MV, mechanical ventilation.

**Table S3 Association of Dementia with Sepsis Mortality in a Complete Dataset**

|  | **Deaths, n. (%)** | | **HR (95%CI)** | **P-value** |
| --- | --- | --- | --- | --- |
|  | **Dementia** | **Non-dementia** |  |  |
| **28-day mortality** |  |  |  |  |
| **Model 1** | 122 (37.8) | 1719 (17.3) | 2.43 (2.02~2.92) | <0.001 |
| **Model 2** |  |  | 1.45 (1.2~1.76) | <0.001 |
| **Model 3** |  |  | 1.26 (1.03~1.52) | 0.022 |
| **90-day mortality** |  |  |  |  |
| **Model 1** | 163(50.5) | 2150(21.6) | 2.73(2.33-3.2) | <0.001 |
| **Model 2** |  |  | 1.61(1.36-1.91) | <0.001 |
| **Model 3** |  |  | 1.37(1.16-1.63) | <0.001 |
| **1-year mortality** |  |  |  |  |
| **Model 1** | 206(28.3) | 2647(26.6) | 3(2.6-3.46) | <0.001 |
| **Model 2** |  |  | 1.72(1.48-2) | <0.001 |
| **Model 3** |  |  | 1.45(1.25-1.69) | <0.001 |

Model 1 was an unadjusted. Model 2 adjusted for age, sex, BMI, smoking, race, and married; Model 3: Model 2+ heart rate, mean arterial pressure, SPO2, hemoglobin, platelets, creatinine, WBC, glucose, potassium, sodium, lactate, SOFA, charlson comorbidity index, MV use (1st 24 h), Vasopressor use (1st 24 h). Abbreviations: CI, confidence interval; HR, hazard ratio; BMI, body mass index; WBC, white blood cell; SOFA, Sequential Organ Failure Assessment; MV, mechanical ventilation.

**Table S4. Summary of the GWAS Data Used in the MR Analyses**

| **Phenotype** | **Ancestry** | **Cases** | **Controls** | **PubMed ID or web source** |
| --- | --- | --- | --- | --- |
| Any dementia | European | 14,367 | 277,526 | www.finngen.fi/en |
| Alzheimer's disease | European | 21,982 | 41,944 | 30820047 |
| Alzheimer's disease  (**Validation**) | European | 39,106 | 48,751 | 35379992 |
| Vascular dementia | European | 2,335 | 360,778 | www.finngen.fi/en |
| Dementia with Lewy bodies | European | 2,591 | 4,027 | 33589841 |
| Sepsis (28day death) | European | 1,896 | 484,588 | NA |

**Table S5. Instrument variables of any dementia**

| **SNP** | **Beta** | **SE** | **EAF** | **P value** | **R^2^** | **F** |
| --- | --- | --- | --- | --- | --- | --- |
| rs679515 | -0.09453 | 0.016647 | 0.807363 | 1.36E-08 | 1.21E-04 | 32.24494758 |
| rs4663105 | 0.096601 | 0.013608 | 0.392317 | 1.26E-12 | 1.89E-04 | 50.38965247 |
| rs9271418 | -0.07662 | 0.013407 | 0.570179 | 1.10E-08 | 1.22E-04 | 32.65893005 |
| rs7982 | 0.079748 | 0.013566 | 0.585796 | 4.14E-09 | 1.29E-04 | 34.55503489 |
| rs7232 | -0.085 | 0.014603 | 0.304356 | 5.86E-09 | 1.27E-04 | 33.88179509 |
| rs138573164 | 0.676284 | 0.032138 | 0.036064 | 2.64E-98 | 1.66E-03 | 442.8126126 |
| rs28615360 | 0.583005 | 0.055894 | 0.011851 | 1.80E-25 | 4.07E-04 | 108.7939409 |
| rs12972156 | 0.554756 | 0.016364 | 0.172672 | 1.00E-200 | 4.29E-03 | 1149.311482 |
| rs1132899 | 0.160352 | 0.013429 | 0.554132 | 7.29E-33 | 5.34E-04 | 142.5713592 |
| rs139713267 | 0.522123 | 0.069153 | 0.008131 | 4.34E-14 | 2.13E-04 | 57.0067951 |
| rs8113128 | 0.310512 | 0.031707 | 0.042759 | 1.20E-22 | 3.59E-04 | 95.90603981 |

**Table S6. Instrument variables of Alzheimer's disease**

| **SNP** | **Beta** | **SE** | **EAF** | **P value** | **R^2^** | **F** |
| --- | --- | --- | --- | --- | --- | --- |
| rs679515 | -0.1508 | 0.0183 | NA | 1.55E-16 | 1.06E-03 | 67.90268008 |
| rs6733839 | 0.1693 | 0.0154 | NA | 4.02E-28 | 1.89E-03 | 120.8534039 |
| rs114812713 | 0.298 | 0.0431 | NA | 4.47E-12 | 7.47E-04 | 47.80401788 |
| rs34665982 | -0.0967 | 0.0166 | NA | 5.80E-09 | 5.31E-04 | 33.93307246 |
| rs9381563 | -0.0821 | 0.0148 | NA | 2.93E-08 | 4.81E-04 | 30.77154455 |
| rs11767557 | -0.1028 | 0.0182 | NA | 1.56E-08 | 4.99E-04 | 31.90287819 |
| rs867230 | 0.1333 | 0.0158 | NA | 3.49E-17 | 1.11E-03 | 71.17582951 |
| rs73223431 | 0.0936 | 0.0153 | NA | 8.34E-10 | 5.85E-04 | 37.42443463 |
| rs11257242 | 0.0841 | 0.0154 | NA | 4.64E-08 | 4.66E-04 | 29.82201349 |
| rs1582763 | -0.1232 | 0.0149 | NA | 1.19E-16 | 1.07E-03 | 68.36523189 |
| rs3851179 | 0.1198 | 0.0148 | NA | 5.81E-16 | 1.02E-03 | 65.5204117 |
| rs12590654 | -0.0906 | 0.0157 | NA | 8.73E-09 | 6.59E-04 | 42.15845143 |
| rs72654445 | -0.5425 | 0.0811 | NA | 2.27E-11 | 5.21E-04 | 33.29994398 |
| rs1081105 | 0.942 | 0.0436 | NA | 1.51E-103 | 6.99E-04 | 44.74496668 |
| rs12151021 | -0.1071 | 0.0169 | NA | 2.56E-10 | 3.66E-03 | 234.7352411 |
| rs111278137 | -0.4735 | 0.0713 | NA | 3.20E-11 | 7.25E-03 | 466.7832241 |
| rs139136389 | -0.4938 | 0.0851 | NA | 6.43E-09 | 6.28E-04 | 40.15983731 |
| rs150685845 | 0.5561 | 0.0645 | NA | 6.62E-18 | 6.89E-04 | 44.10088648 |

**Table S7. Instrument variables of Vascular dementia**

| **SNP** | **Beta** | **SE** | **EAF** | **P value** | **F statistic** |
| --- | --- | --- | --- | --- | --- |
| rs4848625 | -0.19103 | 0.040027 | 0.180189 | 1.82E-06 | 22.77 |
| rs2036927 | 0.170567 | 0.035735 | 0.753147 | 1.81E-06 | 22.78 |
| rs2687963 | 0.162855 | 0.034692 | 0.729031 | 2.68E-06 | 22.03 |
| rs72697948 | 0.727707 | 0.147814 | 0.007065 | 8.52E-07 | 24.23 |
| rs33585 | 0.22737 | 0.049213 | 0.092069 | 3.83E-06 | 21.34 |
| rs115337854 | 0.67904 | 0.144672 | 0.007972 | 2.68E-06 | 22.03 |
| rs35341046 | -0.47142 | 0.089768 | 0.039197 | 1.51E-07 | 27.57 |
| rs1145778 | -0.15141 | 0.030247 | 0.56662 | 5.56E-07 | 25.05 |
| rs2222628 | 0.142127 | 0.030947 | 0.604861 | 4.38E-06 | 21.09 |
| rs12804247 | 0.258143 | 0.054586 | 0.070781 | 2.26E-06 | 22.36 |
| rs7307916 | 0.584547 | 0.127385 | 0.010532 | 4.46E-06 | 21.05 |
| rs9562649 | 0.143581 | 0.030978 | 0.452579 | 3.57E-06 | 21.48 |
| rs8002337 | -0.27239 | 0.058186 | 0.938042 | 2.85E-06 | 21.91 |
| rs9559779 | -0.15215 | 0.030954 | 0.402174 | 8.86E-07 | 24.16 |
| rs117241576 | -0.86985 | 0.18604 | 0.011022 | 2.93E-06 | 21.86 |
| rs75381851 | 0.367894 | 0.077462 | 0.03137 | 2.04E-06 | 22.55 |
| rs62118211 | 0.198832 | 0.043496 | 0.128393 | 4.85E-06 | 20.89 |
| rs2972558 | 0.188833 | 0.036232 | 0.76456 | 1.87E-07 | 27.16 |
| rs429358 | 0.654291 | 0.037017 | 0.176844 | 6.50E-70 | 312.41 |
| rs2423040 | -0.26102 | 0.051753 | 0.103328 | 4.57E-07 | 25.43 |
| rs362044 | 0.158842 | 0.034301 | 0.272671 | 3.64E-06 | 21.44 |

**Table S8. Instrument variables of Dementia with Lewy bodies**

| **SNP** | **Beta** | **SE** | **EAF** | **P value** | **F statistic** |
| --- | --- | --- | --- | --- | --- |
| rs2230288 | 1.06082 | 0.149491 | 0.009064 | 1.28E-12 | 50.34 |
| rs2991959 | -0.62497 | 0.12882 | 0.029799 | 1.23E-06 | 23.52 |
| rs59557467 | 0.263878 | 0.056464 | 0.111622 | 2.96E-06 | 21.83 |
| rs6733839 | 0.22653 | 0.038541 | 0.361559 | 4.16E-09 | 34.53 |
| rs365535 | -0.18746 | 0.040503 | 0.328284 | 3.69E-06 | 21.41 |
| rs6599388 | 0.220396 | 0.039982 | 0.310388 | 3.54E-08 | 30.37 |
| rs7680557 | 0.240262 | 0.037128 | 0.496275 | 9.73E-11 | 41.86 |
| rs10039591 | 0.176484 | 0.038307 | 0.596846 | 4.08E-06 | 21.21 |
| rs117947506 | 0.576119 | 0.121953 | 0.018376 | 2.31E-06 | 22.31 |
| rs2467712 | -0.19826 | 0.038149 | 0.398311 | 2.02E-07 | 27.00 |
| rs10980539 | 0.237362 | 0.05139 | 0.140124 | 3.86E-06 | 21.32 |
| rs11006709 | 0.874906 | 0.189619 | 0.007201 | 3.95E-06 | 21.28 |
| rs1334587 | 0.190678 | 0.040897 | 0.279488 | 3.12E-06 | 21.73 |
| rs79077077 | 0.363941 | 0.075369 | 0.057115 | 1.37E-06 | 23.31 |
| rs8058532 | -0.17071 | 0.03721 | 0.537621 | 4.48E-06 | 21.04 |
| rs16943674 | -0.30349 | 0.065499 | 0.105538 | 3.59E-06 | 21.46 |
| rs117310449 | 0.889565 | 0.16344 | 0.008195 | 5.25E-08 | 29.61 |
| rs769449 | 0.902123 | 0.053821 | 0.100074 | 4.65E-63 | 280.86 |
| rs8101195 | 0.241086 | 0.050483 | 0.81773 | 1.79E-06 | 22.79 |

**Table S9 Heterogeneity and pleiotropy tests for the associations of dementia with sepsis (28day death).**

| **Outcomes** | **Pleiotropy test** | | |  |  | **Heterogeneity test** | | | | | |
| --- | --- | --- | --- | --- | --- | --- | --- | --- | --- | --- | --- |
|  | **MR-Egger** | | |  | **MR-Egger** | | |  | **IVW** | | |
|  | **Intercept** | **SE** | **P** |  | **Q** | **df** | **P** |  | **Q** | **df** | **P** |
| Dementia | -0.012 | 0.025 | 0.633 |  | 12.871 | 8 | 0.116 |  | 13.266 | 9 | 0.150 |
| AD | -0.051 | 0.016 | 0.007 |  | 11.569 | 14 | 0.640 |  | 21.117 | 15 | 0.133 |
| AD（Validation） | -0.010 | 0.011 | 0.353 |  | 70.529 | 55 | 0.077 |  | 69.403 | 54 | 0.077 |
| VD | -0.025 | 0.021 | 0.236 |  | 16.277 | 16 | 0.433 |  | 17.817 | 17 | 0.400 |
| DLB | -0.006 | 0.022 | 0.774 |  | 22.584 | 17 | 0.163 |  | 22.697 | 18 | 0.202 |
| VD, Vascular dementia; AD, Alzheimer's disease; DLB, Dementia with Lewy bodies; IVW, Inverse Variance Weighted; df, degree of freedom; MR, Mendelian randomization; Q, heterogeneity statistic. P, P value. | | | | | | | | | | | |

**
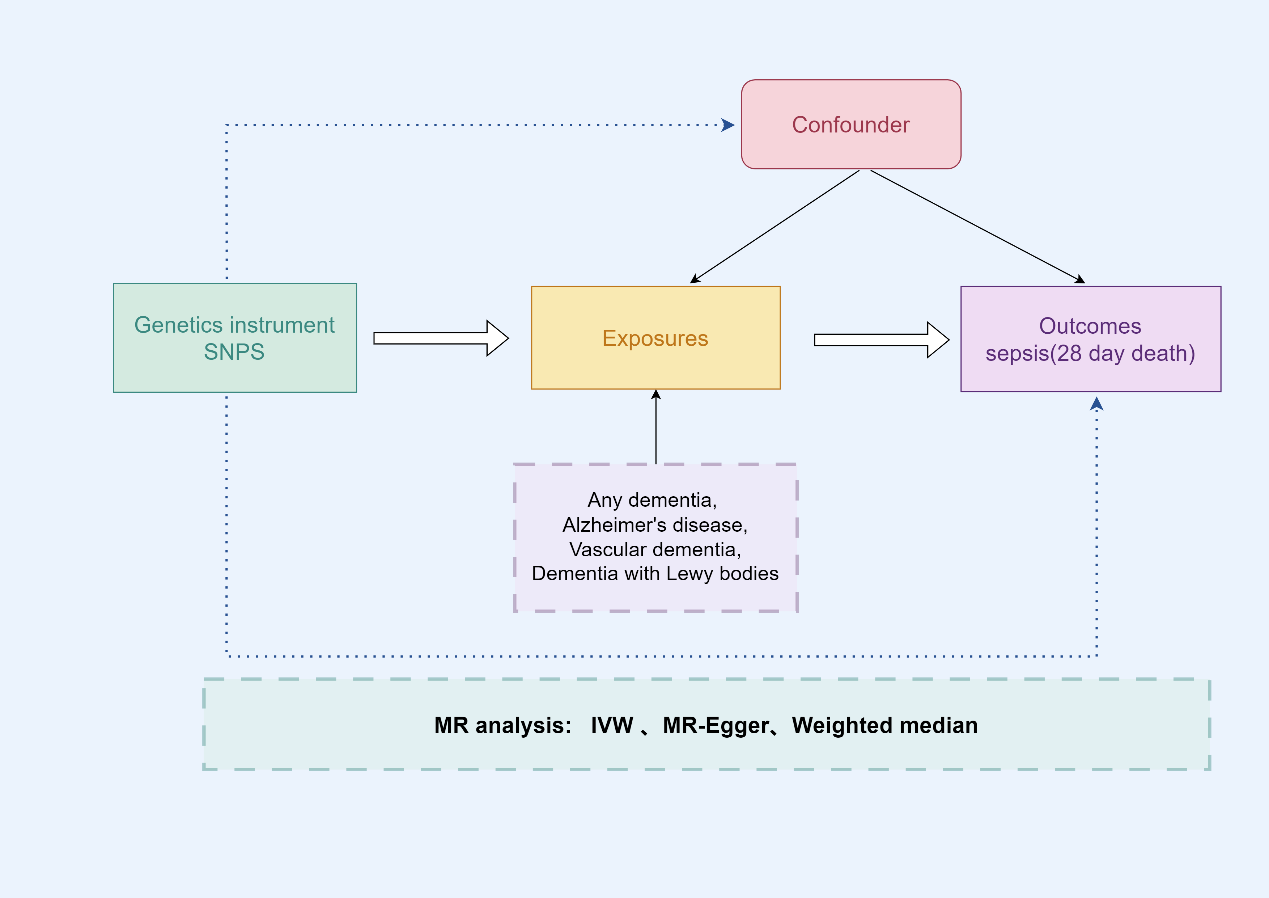
**

**FigureS1 An overview of MR analysis.** SNPs, single-nucleotide polymorphisms; MR, Mendelian randomization; IVW, inverse variance weighted.

**
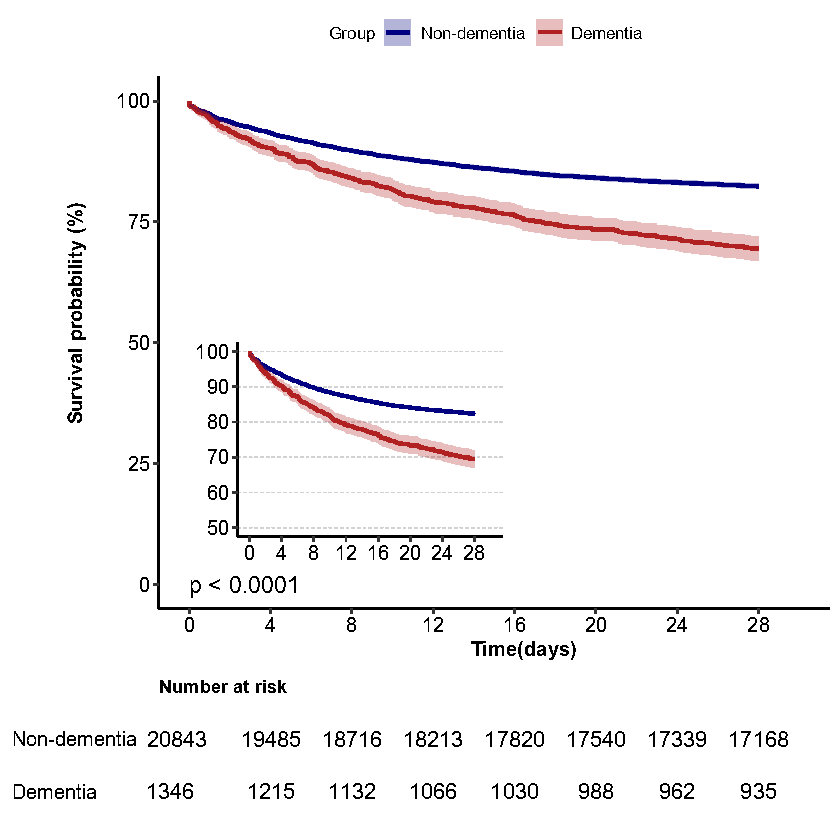
**

**Figure S2 Kaplan–Meier analysis for 28-day survival probability in patients with sepsis grouped by dementia.**

**Legend**: This Kaplan-Meier curve illustrates the survival probability over time for the dementia group and the non-dementia group during the 28-day follow-up period. The survival curves are represented in blue for the non-dementia group and red for the dementia group. The inset panel in the lower-left corner provides a magnified view of the curves, aimed at offering a clearer presentation of survival rate trends within this time frame. The trend in survival rates remains consistent with the main figure; the panel simply enlarges the area to facilitate better observation of differences between the two groups.

**
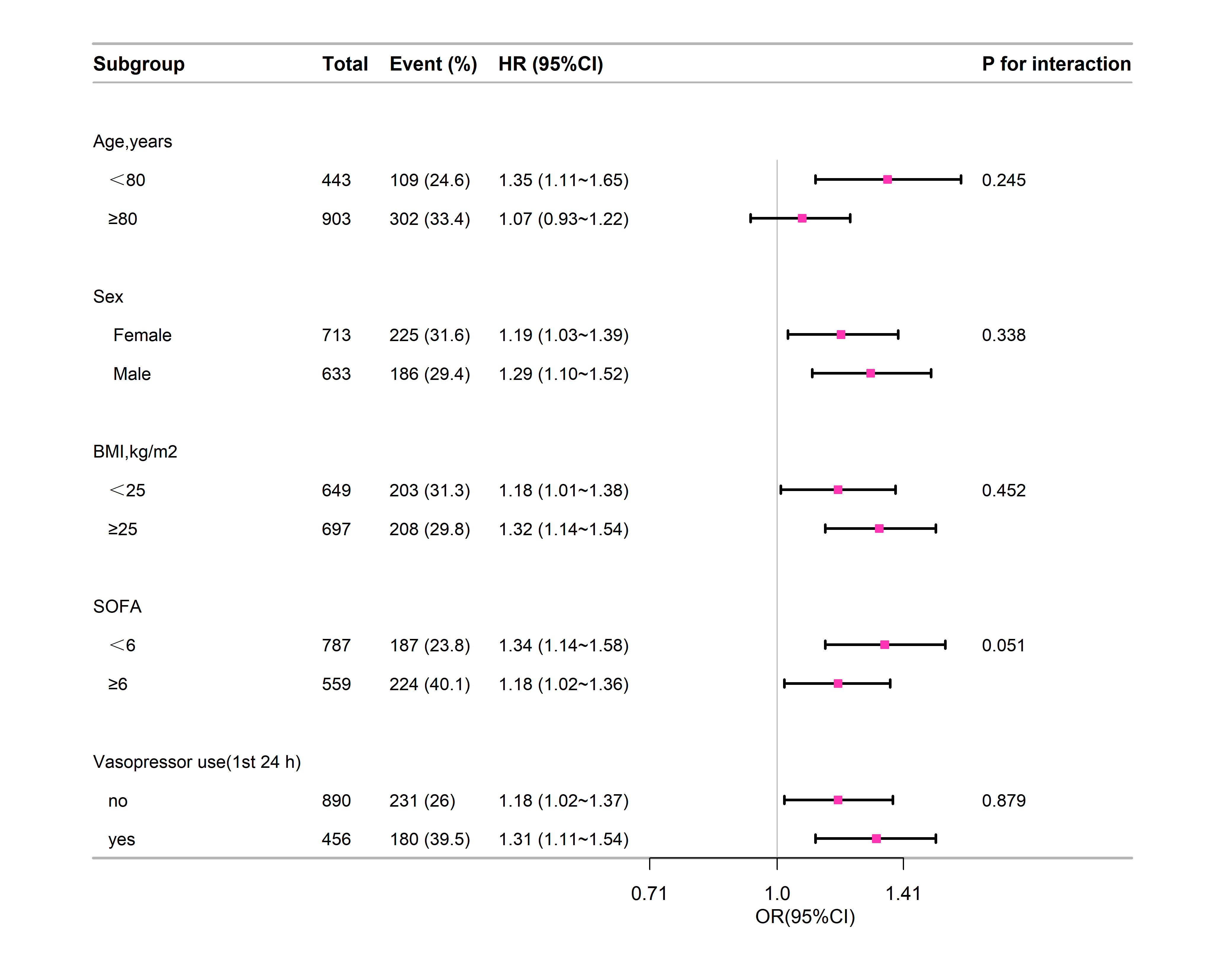
**

**Figure S3 Subgroup analyses for the association of dementia with 28-day mortality.** HR: Hazard ratio, CI: confidence interval; BMI, body mass index; SOFA, Sequential Organ Failure Assessment.
